# An exploratory study on the association between serotonin and sleep breathing disorders

**DOI:** 10.1038/s41598-023-38842-y

**Published:** 2023-07-21

**Authors:** Mieszko Wieckiewicz, Helena Martynowicz, Gilles Lavigne, Frank Lobbezoo, Takafumi Kato, Efraim Winocur, Joanna Wezgowiec, Dariusz Danel, Anna Wojakowska, Grzegorz Mazur, Joanna Smardz

**Affiliations:** 1 Department of Experimental Dentistry, Wroclaw Medical University, Wroclaw, Poland; 2 Department and Clinic of Internal Medicine, Occupational Diseases, Hypertension and Clinical Oncology, Wroclaw Medical University, Wroclaw, Poland; 3 Faculty of Dental Medicine, Universite de Montreal, CIUSSS Nord Ile de Montreal and CHUM, Montreal, Canada; 4 Department of Orofacial Pain and Dysfunction, Academic Centre for Dentistry Amsterdam (ACTA), University of Amsterdam and Vrije Universiteit Amsterdam, Amsterdam, The Netherlands; 5 Department of Oral Physiology, Osaka University Graduate School of Dentistry, Suita, Japan; 6 Department of Oral Rehabilitation, The Maurice and Gabriela Goldschleger School of Dental Medicine, Tel Aviv University, Tel Aviv, Israel; 7 Department of Anthropology, Hirszfeld Institute of Immunology and Experimental Therapy, Polish Academy of Sciences, Wroclaw, Poland

**Keywords:** Sleep disorders, Sleep disorders

## Abstract

This exploratory observational study aimed to evaluate whether the blood levels of serotonin and enzymes involved in serotonin synthesis are associated with sleep breathing parameters. A total of 105 patients were included in this study, who were subjected to single-night polysomnography with simultaneous audio–video recordings. Peripheral blood samples were collected to estimate the serum levels of serotonin, tryptophan hydroxylase 1 (TPH1), and aromatic l-amino acid decarboxylase (AADC). Results showed a negative correlation between blood serotonin levels, and oxygen desaturation index (ODI) (*p* = 0.027), central apnea (*p* = 0.044) and obstructive apnea (OA) (*p* = 0.032) scores. Blood TPH1 levels were negatively correlated with average (*p* = 0.003) and minimal saturation (*p* = 0.035) and positively correlated with apnea–hypopnea index (*p* = 0.010), OA (*p* = 0.049), and hypopnea index (*p* = 0.007) scores. A tendency to sleep-disordered breathing seemed to co-occur with lower blood serotonin and higher TPH1 levels.

Clinical Trial Registration :www.ClinicalTrials.gov, identifier NCT04214561

## Introduction

Sleep-disordered breathing (SDB) refers to a wide spectrum of common sleep-related conditions, such as sleep-related hypoventilation, hypoxemia, snoring, and obstructive and central sleep apnea^1^. It is characterized by repetitive sleep-related respiratory episodes and intermittent hypoxia^1,2^. Patients with SDB can experience daytime symptoms such as sleepiness and excessive somnolence^3,4^, depressed mood^5^, irritability^5,6^, and cognitive dysfunction^6^, which can adversely affect their health-related quality of life^6–8^. These effects of SDB are attributable to both apnea-associated oxygen desaturation and sleep disturbance^9–11^. Ultimately, SDB can lead to a number of medical and social problems, such as an increase in the number of car accidents, hypertension, cognitive impairment, impotence, and even early death^1–10^. Obstructive sleep apnea (OSA) is the most common and most prevalent type of SDB. It is grossly undiagnosed, affecting one billion of the adult population worldwide^1^. Obesity, advanced age, and male gender are the primary risk factors for OSA. Due to the increased risk of stroke and heart attack, untreated OSA can lead to higher levels of comorbidities and mortality compared with other SDB conditions^1,10^.Serotonin, also known as 5-hydroxytryptamine, is a monoamine neurotransmitter that also acts as the “happiness” hormone. It is a monoamine responsible for neurotransmission in both central and peripheral nervous systems. Serotonin plays various roles in the human body: it influences learning, memory, body temperature, sleep, sexual behavior, hunger, and mood^12–17^. Serotonin is synthesized from L-tryptophan by tryptophan hydroxylase 1 (TPH1) and aromatic l-amino acid decarboxylase (AADC). Being the initial enzyme in serotonin synthesis, TPH1 is irreversibly inactivated by nitric oxide^18–20^ and inhibited by hypoxia^21^. Serotonin also plays an important role in respiratory function. Reduction in serotonin biosynthesis may be attributable to the complex influence of nitric oxide and hypoxia^21^. Furthermore, serotonergic neurotransmission modulates the activity of brainstem cardiorespiratory circuits^21^. The first reports on the influence of serotonin on respiratory functions date back to 1953. Studies reported the involvement of intravenous serotonin injections in the central regulation of breathing. It caused apnea episodes in cats^22^. However, in similar experiments involving dogs, breathing stimulation was observed^23^. In humans, the same procedure resulted in a direct effect of serotonin on the receptors located on the arterial side of systemic circulation^21^. Some studies showed that the application of serotonin into medullary respiratory neurons enhanced their excitation^24^. Besides the neuromodulatory role in the central respiratory system, serotonin plays an important role in blood gas homeostasis. Serotonin neurons located near the arteries entering the brainstem may contribute to the adaptation of the central respiratory drive to environmental changes by detecting the changes in arterial CO2^21^. As blood platelets are considered a useful peripheral model to investigate the central serotoninergic mechanisms^25,26^, some of the research aimed to evaluate weather there is a relationship between serum serotonin level and apnea. Murugesan et al. reported that significant seizure-related increases in serum serotonin levels were associated with a lower incidence of seizure-related breathing dysfunction^27^. What is more, some research has already shown that two polymorphisms of the serotonin transporter gene—variable number of tandem repeats and linked polymorphic regions—are important factors involved in the pathogenesis of OSA. Specific allele combinations can be associated with a higher prevalence of OSA, a higher apnea–hypopnea index (AHI), and a longer time during sleep with oxygen desaturation^28^. Furthermore, some studies have reported the effect of OSA therapy on increasing blood serotonin levels^29^. It is worth emphasizing that the production of serotonin would not occur without the participation of two key enzymes: TPH1 and AADC^14–17^, which may also play a potential role in the aforementioned dependencies.

It should be also emphasized that, among all putative factors associated with human mental well-being, serotonin is one of the leading factors^12^. Moreover, several pharmacological approaches target the serotonin pathway in the treatment of mental disorders such as anxiety, depression, and mania^17^. As psychoemotional disturbances such as depression and anxiety were observed in about one-third of the individuals with OSA^30,31^, the question arises whether these conditions may, at some stage, be dependent on the serotonin pathway.

Nevertheless, the question of whether SDB is associated with blood serotonin levels and enzymes responsible for serotonin synthesis remains unanswered. Therefore, this study aimed to evaluate whether the blood levels of serotonin and TPH1 and AADC involved in serotonin synthesis are associated with sleep breathing parameters.

## Methods

### Participants

The participants were adult patients hospitalized in the Department and Clinic of Internal Medicine, Occupational Diseases, Hypertension and Clinical Oncology, Wroclaw Medical University in 2020 and 2021. This study was carried out in accordance with the guidelines of the Declaration of Helsinki and was approved by the Ethical Committee of Wroclaw Medical University (ID: KB-794/2019). All participants provided written informed consent, and the information regarding clinical trial registration can be found at www.ClinicalTrials.gov (identifier: NCT04214561).

### Inclusion criteria

The inclusion criteria were the following: age ≥ 18 years, clinical suspicion of SDB, and willingness to participate in the study .

### Exclusion criteria

The following parameters served as the exclusion criteria: severe systemic disorders and diseases (including genetic disorders and based on the existing hospital protocols); neurological disorders and/or neuropathic pain; active inflammation; active malignancy; severe mental disorders and significant mental (including genetic) disabilities; pregnancy and confinement; treatment with or addiction to any analgesic agents and/or drugs that can affect the functions of the nervous system, muscles, and respiratory system and sleep; and the lack of agreement to participate in the study .

### Recruitment

The participants were recruited among the patients of the Outpatient Clinic of Temporomandibular Disorders, Department of Experimental Dentistry, Wroclaw Medical University. We used a convenience sampling method, meaning all patients who were available at the clinic and met inclusion criteria were asked to participate in the study.

During the recruitment procedure, the patients were subjected to a thorough medical interview and an intra- and extraoral examination following the Diagnostic Criteria for Temporomandibular Disorders^32^ via self-reporting (including reporting by bed partner if he/she was available), and the signs and symptoms of SDB were assessed by two experienced dentists. The patients were additionally asked standard questions about potential problems with breathing during sleep (Do you or your partner noticed that you snore?; Do you or your partner noticed your breathing interruption during sleep?; Do you have excessive daytime sleepiness and fatigue not explained by other factors?; Are you obese?)^33^. Those who were suspected to be at risk of SDB, in accordance with the Third Edition of the International Classification of Sleep Disorders by the American Academy of Sleep Medicine^34^, were subjected to single-night audio-video polysomnography (avPSG) diagnostics.

Based on the 2013 American Academy of Sleep Medicine standard criteria for sleep scoring^35^, comparative analyses showing the lack, the presence, and the severity of SDB and OSA were carried out in the following groups:

1. ODI < 5 and ODI ≥ 5 ,
2. AHI < 5 and AHI ≥ 5 ,
3. AHI < 15 and AHI ≥ 15 ,
4. AHI < 30 and AHI ≥ 30 , and
5. average saturation < 93% and average saturation ≥ 93% .

According to the American Academy of Sleep Medicine, AHI and ODI were categorized into mild (5–15 events/h), moderate (15–30 events/h), and severe (> 30 events/h) . Average saturation values < 93% showed the risk of SDB^35^.

### Polysomnography (PSG)

avPSG was carried out using NoxA1 (NOX Medical, Reykjavík, Iceland) in the Sleep Laboratory, Department and Clinic of Internal Medicine, Occupational Diseases, Hypertension, and Clinical Oncology, Wroclaw Medical University. The recordings were carried out from 10:00 pm to 06:00 am .

During avPSG, the following standard elements were evaluated: electroencephalographic, electrocardiographic, electrooculographic, and electromyographic recordings from the chin area and bilaterally from the masseter muscles; recordings of abdominal and thoracic breathing activity; body position; and audio–video recordings. The saturation level, pulse, and plethysmographic data were recorded using a NONIN WristOx2 3150 pulse oximeter (Nonin Medical Inc., Plymouth, MN, USA). The entire avPSG recording was reconstructed using the Noxturnal software (Nox Medical, Reykjavík, Iceland). All avPSG recordings were scored and analyzed in 30-s epochs by a qualified and experienced physician in accordance with the AASM Manual for the Scoring of Sleep and Associated Events, Version 2.4^34,35^.

The following breathing parameters were measured, among others: the AHI, oxygen desaturation index (ODI), obstructive apnea (OA), central apnea (CA), hypopnea index (HI), average and minimal saturation, and average desaturation decline^34,35^.

### Assessment of serotonin and enzyme levels

To analyze the levels of serotonin and enzymes involved in the serotonin synthesis pathway, namely TPH1 and AADC, 4 mL of peripheral blood was collected between 8:00 and 9:00 am from an antecubital vein from each alcohol- and medication-free patient using the Vacutainer® (Becton Dickinson, Franklin Lakes, NJ, USA) blood sampling system. The blood samples, which were collected into anticoagulant-free tubes, were centrifuged at 4500 × *g* for 10 min to obtain serum. The resulting serum samples were transferred to 1.5-mL Eppendorf tubes (Eppendorf, Hamburg, Germany), frozen, and stored at − 80 °C until further analysis. The methods of serotonin and enzyme levels assessment were based on the methods described in our previous studies^36,37^.

#### Serotonin serum level measurement

The serum level of serotonin was determined using a commercially available enzyme-linked immunosorbent assay (ELISA) kit (ref. RE59121; IBL International GmbH, Hamburg, Germany), with a sensitivity of 1.5 ng/mL and a detection range of 23–355 ng/mL. The ELISA procedure followed the basic principle of competitive ELISA and was carried out according to the manufacturer’s instructions (the version with overnight incubation). Optical density (OD) was measured using a microplate reader (GloMax® Discover, Promega, Madison, USA) at 405 nm, with the reference wavelength at 600 nm. GraphPad Prism (GraphPad Software, San Diego, USA) was used for standard curve fitting. The concentrations of the samples were then quantified. Each sample was measured in triplicate, and the mean serum levels of serotonin (ng/mL) were calculated for each patient based on the standard curve fitted^36,37^.

#### TPH1 serum level measurement

The serum level of TPH1 was evaluated using a commercially available ELISA kit (ref. E-EL-H5313; Elabscience, Houston, TX, USA), with a sensitivity of 0.19 ng/mL and a detection range of 0.31–20 ng/mL. The procedure was carried out following the basic principle of Sandwich ELISA, according to the manufacturer’s instructions. After optimizing the laboratory procedure, the serum samples were diluted tenfold with the sample diluent to match the measured values to the detection range of the ELISA kit. OD was measured using a microplate reader (Multiskan GO; Thermo Fisher Scientific, Waltham, MA, USA) at 450 nm. GraphPad Prism (GraphPad Software, San Diego, CA, USA) was used for standard curve fitting, and then, the concentrations of the samples were quantified. Each sample was measured in triplicate, and the mean serum levels of TPH1 (ng/mL) were calculated for each patient based on the standard curve fitted^36,37^.

#### AADC serum level measurement

The serum level of human AADC was measured using a commercially available ELISA kit (ref. ELH-DDC-1; RayBiotech, Peachtree Corners, GA, USA), with a sensitivity of 34 pg/mL and a detection range of 34–8000 pg/mL. The procedure was based on Sandwich ELISA and carried out according to the manufacturer’s instructions. The serum samples were diluted twofold using the assay diluent. OD was measured using a microplate reader (GloMax® Discover; Promega, Madison, WI, USA) at 450 nm. GraphPad Prism (GraphPad Software, San Diego, CA, USA) was used for standard curve fitting, and then, the concentrations of the samples were quantified. Each sample was measured in triplicate, and the mean serum levels of AADC were calculated (pg/mL) for each patient based on the standard curve fitted^36,37^.

### Data analysis

The obtained data were analyzed using the TIBCO Software Inc. (2017), Statistica (data analysis software system), version 13 (http://statistica.io). The results were considered statistically significant at *p* < 0.05. The data distribution and potential deviations from the normal distribution were analyzed using the Shapiro–Wilk test (*p* < 0.05 indicating nonnormal distribution). Between-group differences were analyzed using the Mann–Whitney U test. Correlation analysis was carried out using Spearman’s rank correlation test.

The statistical approach was based on a series of correlation analyses and between-group comparisons. We used these relatively simple methods due to the preliminary nature of our study aimed at the initial exploration of the association between serotonin and sleep breathing disorders. This approach, however, is prone to inflation of type I errors and premature rejections of true null hypotheses. One of the most common ways to address this problem is to adequately adjust the statistical significance level alpha (e.g., using Bonferroni adjustments). Nonetheless, in many cases, this approach is too conservative and may entail unnecessary rejections of alternative hypotheses, leading to the oversight of actual phenomena (i.e., type II error). Therefore, especially in exploratory medical research (such as the present study), it has been suggested that the reduction in the significance level should be used with caution or even abandoned^38,39^. Correspondingly, in the present study, unadjusted statistical significance levels were reported while underlining the exploratory characteristics of the findings.

## Results

A total of 105 patients were included in this study (80 women and 25 men). All patients were Caucasians aged 18–63 years, with a mean ± standard deviation (SD) age of 33.43 ± 10.8 years. The average body mass index (BMI) ± standard deviation (SD) of the patients was 22.60 ± 3.47. Of the included patients, 82 (78.1%) showed normal weight (BMI < 25), 17 patients (16.19%) were overweight (BMI ≥ 25 and < 30), and 6 (5.71%) patients were obese (BMI ≥ 30). A total of 88 participants presented AHI < 5 (no sleep apnea), 11 participants presented AHI 5–15 (mild sleep apnea), three participants presented AHI 15–30 (moderate sleep apnea), and three participants presented AHI > 30 (severe sleep apnea). The descriptive statistics of studied parameters are presented in Table 1.

**Table 1:** Descriptive statistics of all studied parameters.

	N females	N males	Median	Quartile 1	Quartile 3	Shapiro–Wilk (normality)
Age	80	25	33,0	26,00	41,0	W = .95918. p = .0026
AHI	80	25	2	0,8	3,7	W = .46729, *p* < .0001
ODI	80	25	2	1	3,7	W = .46600, *p* < .0001
OA	80	25	0	0	0,1	W = .19513, *p* < .0001
CA	80	25	0,1	0	0,4	W = .69168, *p* < .0001
HI	80	25	1,4	0,5	2,9	W = .49016, *p* < .0001
Average saturation	80	25	95,4	94,8	96	W = .87995, *p* < .0001
Minimal saturation	80	25	91	88	93	W = .66522, *p* < .0001
Average desaturation decline	77	25	3,1	3	3,5	W = .76897, *p* < .0001
Average serotonin level [ng/mL]	80	25	104,9	72,8	129,7	W = .90960, *p* < .0001
Average AADC level [pg/mL]	80	25	1117	754,6	1581,6	W = .80587, *p* < .0001
Average TPH1 level [ng/mL]	80	25	78,2	40,3	149,6	W = ,78,568, *p* < *.*0001

Apnea–hypopnea index (AHI), oxygen desaturation index (ODI), obstructive apnea (OA), central apnea (CA), hypopnea index (HI), aromatic l-amino acid decarboxylase (AADC), tryptophan hydroxylase 1 (TPH1) ; *p* < 0.05 indicates nonnormal distribution.

### Sleep breathing parameters and serotonin levels

Average blood serotonin levels [ng/mL] were negatively correlated with ODI, CA, and OA scores. Other sleep breathing parameters were not significantly correlated with serotonin levels (Table 2).

**Table 2:** Associations between average blood serotonin levels and breathing parameters.

Parameter	*r* Spearman	*p*-Value
AHI	− 0.155	0.114
ODI	− 0.215	0.027
OA	− 0.210	0.032
CA	− 0.197	0.044
HI	− 0.155	0.114
Average saturation	0.079	0.424
Minimal saturation	0.162	0.099
Average desaturation decline	− 0.160	0.109

Apnea–hypopnea index (AHI), oxygen desaturation index (ODI), obstructive apnea (OA), central apnea (CA), hypopnea index (HI).

### Sleep breathing parameters and TPH1 levels

Average blood TPH1 levels [ng/mL] were negatively correlated with the average and minimal saturation and positively correlated with AHI, OA, and HI scores. Other sleep breathing parameters were not significantly correlated with TPH1 levels (Table 3).

**Table 3:** Associations between average blood tryptophan hydroxylase 1 levels and breathing parameters.

Parameter	*r* Spearman	*p*-Value
AHI	0.252	0.010
ODI	0.192	0.050
OA	0.193	0.049
CA	− 0.028	0.777
HI	0.264	0.007
Average saturation	− 0.283	0.003
Minimal saturation	− 0.206	0.035
Average desaturation decline	0.097	0.333

Apnea–hypopnea index (AHI), oxygen desaturation index (ODI), obstructive apnea (OA), central apnea (CA), hypopnea index (HI).

### Sleep breathing parameters and AADC levels

Average blood AADC levels [pg/mL] were negatively correlated with HI scores and positively correlated with OA and average saturation. Other sleep breathing parameters were not statistically significantly correlated with AADC levels (Table 4).

**Table 4:** Associations between average blood aromatic l-amino acid decarboxylase levels and breathing parameters.

Parameter	*r* Spearman	*p*-Value
AHI	− 0.157	0.110
ODI	− 0.162	0.098
OA	0.295	0.002
CA	0.133	0.175
HI	− 0.213	0.029
Average saturation	0.199	0.041
Minimal saturation	0.147	0.135
Average desaturation decline	− 0.018	0.860

Apnea–hypopnea index (AHI), oxygen desaturation index (ODI), obstructive apnea (OA), central apnea (CA), hypopnea index (HI).

### Comparisons in groups

Since the data obtained did not meet the assumptions for parametric tests, nonparametric equivalents (Mann–Whitney U test for between-group comparisons and Spearman rank correlation coefficient for association analyses) were used in this study. The Mann–Whitney U analysis showed higher average TPH1 blood levels in the groups of AHI ≥ 15, AHI ≥ 30, and average saturation < 93%. It also showed lower average blood serotonin levels in the group of ODI ≥ 5. No significant differences between groups were observed in comparisons of other studied parameters (Table 5).

**Table 5:** Comparisons by severity cutoff of groups of sleep breathing parameters and serotonin findings.

	Mean ± SD		U	Z	*p*-Value
	AHI < 5	AHI ≥ 5			
	*N* = 88	*N* = 17			
TPH1	109.15 ± 119.233	155.75 ± 122.988	559.00	− 1.640	0.101
AADC	1384.70 ± 915.643	1047.40 ± 559.005	593.00	1.344	0.179
Serotonin	110.01 ± 55.773	93.41 ± 51.520	640.00	0.935	0.350

	AHI < 15	AHI ≥ 15			
	*N* = 99	*N* = 6			
TPH1	109.88 ± 118.851	229.15 ± 101.851	108.00	− 2.602	**0.009**
AADC	1343.09 ± 896.472	1115.65 ± 319.490	290.00	0.090	0.928
Serotonin	108.52 ± 55.552	87.60 ± 49.218	239.00	0.794	0.427

	AHI < 30	AHI ≥ 30			
	*N* = 102	*N* = 3			
TPH1	112.45 ± 119.378	260.92 ± 55.754	34.00	− 2.279	**0.023**
AADC	1335.88 ± 885.440	1133.14 ± 358.859	153.00	0.010	0.992
Serotonin	107.55 ± 55.752	99.67 ± 38.078	142.00	0.202	0.840

	ODI < 5	ODI ≥ 5			
	*N* = 88	*N* = 17			
TPH1	109.20 ± 118,911	155.47 ± 124,787	574.00	− 1.509	0.131
AADC	1380.75 ± 916,682	1067.84 ± 562,370	613.00	1.170	0.242
Serotonin	112.38 ± 56,053	81.12 ± 43,114	515.00	2.023	**0.043**

	AS < 93%	AS ≥ 93%			
	*N* = 9	*N* = 96			
TPH1	226.64 ± 175.134	106.38 ± 109.806	199.00	− 2.661	**0.008**
AADC	1091.39 ± 395.088	1352.47 ± 904.679	399.00	0.372	0.710
Serotonin	87.06 ± 35.832	109.22 ± 56.448	322.00	1.253	0.210

Apnea–hypopnea index (AHI), oxygen desaturation index (ODI), average oxygen saturation (AS), standard deviation (SD), tryptophan hydroxylase 1 (TPH1), aromatic l-amino acid decarboxylase (AADC).

## Discussion

This study aimed to evaluate whether the blood levels of serotonin and TPH1 and AADC^14–17^ involved in serotonin synthesis are associated with sleep breathing parameters. The most important finding of the present study was that average blood serotonin levels [ng/mL] were negatively correlated with ODI, CA, and OA scores. Blood TPH1 levels [ng/mL] were negatively correlated with the average and minimal saturation and positively correlated with AHI, OA, and HI scores. Furthermore, a lower average blood serotonin level was observed in the group of ODI ≥ 5. Although statistical significance was observed, other group comparisons were not discussed due to the large disproportion in the sample size of specific groups, which is a potential bias in accordance with the conclusions of the study. Also, the female-biased sex ratio of the final study sample and the underrepresentation of men’s patients require caution when interpreting and generalising the observed results.

As mentioned in the Introduction section, serotonin neurotransmission was reported to potentially influence SDB associated with hypoxia. Two polymorphisms of the serotonin transporter gene—variable number of tandem repeats and linked polymorphic regions—are crucial factors in OSA's pathogenesis. Specific allele combinations can be associated with a higher prevalence of OSA, a higher AHI, and a longer time during sleep with oxygen desaturation^28^. Tendency to SDB can be suspected in accordance with both the most used sleep breathing parameter, i.e., AHI, and ODI, which could be even more relevant in predicting the tendency to SDB in patients without apnea^40^. Nevertheless, studies investigating how the levels of serotonin and enzymes involved in serotonin synthesis affect SDB are not available in the literature.

In the present study, lower serotonin levels were shown to be associated with higher ODI scores and an increase in the number of obstructive and central sleep apnea episodes. Furthermore, in general, individuals with ODI ≥ 5 showed lower serotonin levels compared with those with ODI < 5. The potential cause of this result may be the fact that hypoxia accompanying the described events is considered the cause of the reduction in serotonin biosynthesis^21,41,42^. Moreover, one of the available studies showed that the proper treatment of SDB with the aim of hypoxia reduction reported a significant improvement in blood serotonin levels^29^. In addition, drugs whose action is based on the reuptake of serotonin are shown to be effective in some cases in reducing CA^43^. Thus, the results of previous studies support the findings of the present study.

In addition, in the present study, lower levels of TPH1 were correlated with a higher average and minimal saturation and lower AHI, OA, and HI scores. As TPH1 was reported to be inhibited by hypoxia and irreversibly inactivated by nitric oxide, it is no surprise that in individuals with a tendency to SDB (higher AHI, OA, and HI and lower saturation), TPH1 levels are higher as a part of the potential adaptation process^21^. However, it should not be forgotten that TPH1 in the human body is mostly present intracellularly^44^.

Based on the aforementioned findings, the factors contributing to the levels of AADC seem quite surprising. Analogous to TPH1, higher levels of AADC were associated with higher OA scores, but not with lower average saturation and higher HI scores. This relationship needs to be studied more thoroughly due to differences in the results concerning TPH1 and AADC.

Interestingly, some parameters used in this study were more consistent across outcome measures than others. The most consistent parameter was OA, higher values of which were associated with lower serotonin and higher TPH1 and AADC levels. OA is characterized by complete or partial obstruction episodes of the upper airway, which leads to a huge reduction in or even the absence of airflow through the airway during sleep. This obstruction is triggered by the loss of upper airway dilator muscle tone and the inadequate compensatory response of the muscles in the anatomically compromised airway^45^. Serotonin has excitatory effects on hypoglossal motoneurons, but during sleep, its delivery to upper airway dilator motor neurons is reduced. Thus, a decreased blood serotonin level could be a potential factor for the increased risk of the loss of upper airway muscle tone^46,47^. Furthermore, hypoxia that accompanies apnea has been reported to negatively affect peripheral serotonin uptake and degradation in some living organisms^48^. Therefore, the findings of the present issue should be investigated from two perspectives: in terms of reduced serotonin levels as a potential cause, and a potential effect of OA. In light of the results showing the relationship between OA and increased levels of enzymes involved in serotonin biosynthesis, studies are available that indirectly suggest that the increased level of TPH1 may also be the effect of hypoxia^49^. This could also be observed in the results of the present study.

As serotonin is responsible for human mental well-being, it should be emphasized that the possible cause-and-effect sequence, including airway obstruction, subsequent hypoxia, and reduced serotonin levels with a simultaneous increase in the levels of enzymes involved in its synthesis, is gaining significant interest in the psychoemotional aspect as well. OSA was previously reported to be associated with depression and anxiety^50^. Furthermore, depression could potentially be considered one of the symptoms of OSA^51^. Therefore, the question arises whether the aspects of possibly disturbed serotonin synthesis and its reduced level in patients with hypoxia related to OSA have an impact on the development of psychoemotional disorders. If so, the important question of whether good sleep breathing is one of the aspects of a “good and happy” life needs further research. Also, further research requires consideration of other mechanisms that increase the risk of depression in SDB individuals, such as brain-derived neurotrophic factor signaling pathway^52,53^.

To the best of our knowledge, no study has reported the measurement of both blood serotonin levels and enzyme levels involved in serotonin synthesis in the aspect of tendency to SDB using the most objective SDB diagnostic method (avPSG). In addition, this study involved a large group of participants (*n* = 105), which is extremely important in light of the limited access to PSG in many countries. Another strength of this study is the fact that it also included participants who had not yet developed factors that independently contribute to SDB (advanced age, obesity, systemic disorders). Despite showing promising results that confirmed the involvement of the serotoninergic pathway in the pathogenesis of SDB, the present study has several limitations. First, only one-night avPSG examinations were conducted because of the restrictions of the Polish healthcare system. Second, this study has an exploratory nature, which means that the results need to be supported by further studies. Third, the majority of the participants were female, and the number of OSA patients is low, with no mood and comorbidities data available. Fourthly, in the process of qualifying participants for the study, validated questionnaires useful in the diagnosis of OSA were not used. Future studies need to be carried out using questionnaires on well-being, mood, and anxiety or, as a proof of concept, serotonin medication in individuals with OSA and mood alterations. Nevertheless, due to the use of objective diagnostic methods and the possibility of application in patients at risk of SDB, the results of the present study show huge potential for further research and clinical purposes.

## Conclusions

The results of this study seem to further support the association of blood serotonin levels and TPH1 blood levels with the increased values of sleep breathing parameters related to a higher probability of SDB. The results show that the tendency to SDB may be related to decreased serotonin and increased TPH1 blood levels. Since the results seem to be most consistent for OA, it seems to be associated also with the increased blood levels of both TPH1 and AADC. Due to the exploratory nature of the study, these relationships need to be confirmed in further clinical studies involving larger sample sizes and individuals presenting more advanced SDB. However, it is worth emphasizing that the results of the present study have a high clinical and diagnostic value. Due to the poor availability of polysomnographic sleep assessment, symptoms indicating low serotonin levels (depressed mood, sleeping disturbances) based on simple blood tests, while also meeting some of the other SDB risk criteria, may suggest the need to conduct more detailed SDB diagnostics. This will help identify individuals at risk of SDB and implement prevention and treatment at an earlier stage.

## Data Availability

The data supporting the conclusions of this study are present in the paper. The raw datasets used for the analysis within the present study are available from the corresponding author upon reasonable request.
